# Supplementary figures and images for: Expression and purification of a functional heteromeric GABAA receptor for structural studies
Source: PLoS One. 2018 Jul 20;13(7):e0201210. doi: 10.1371/journal.pone.0201210 (PMC6054424; doi:10.1371/journal.pone.0201210)

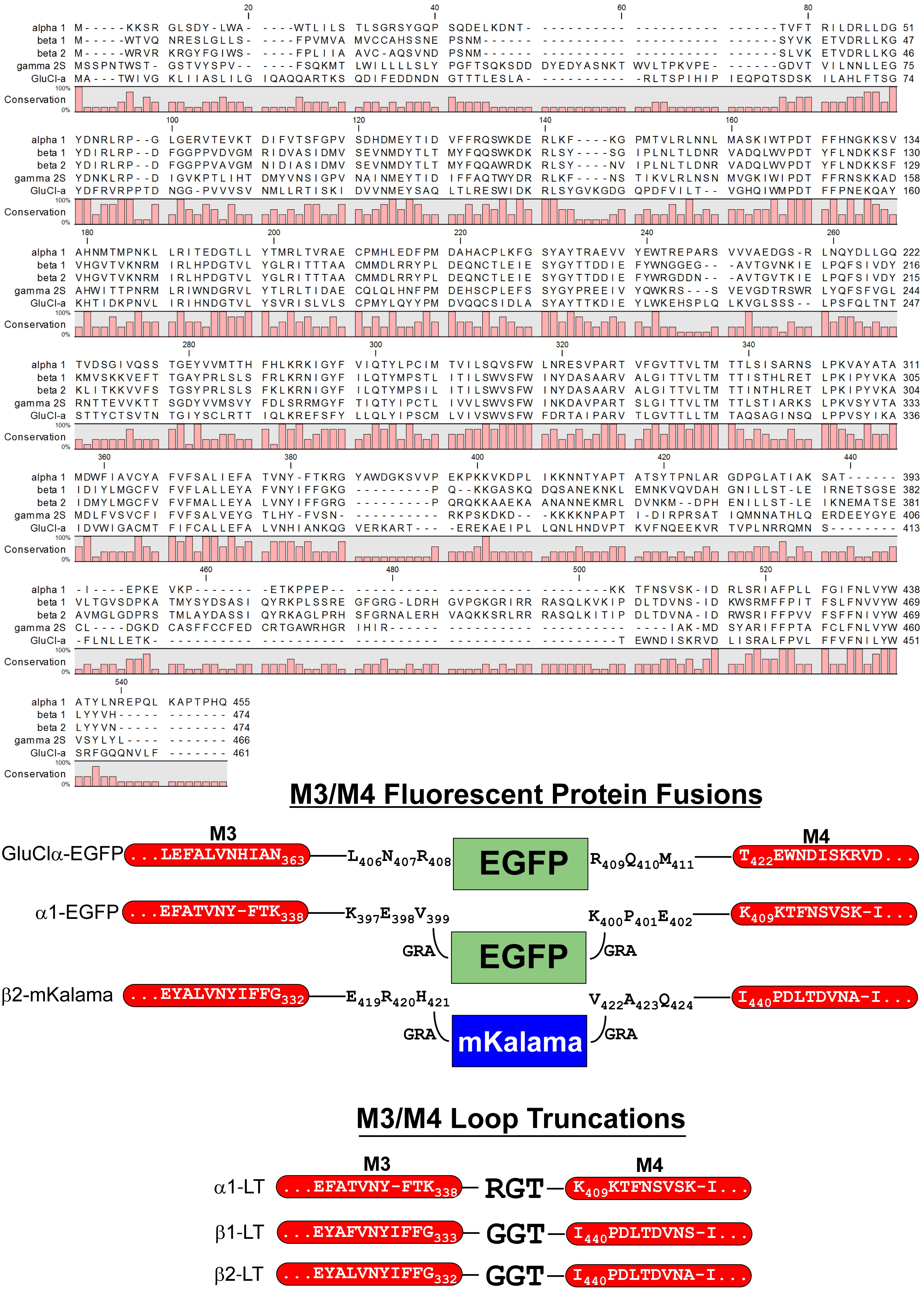

Supplement: S1 Fig — Alignment of GABAA subunit genes used in this study with GluClα highlights regions of sequence variation found in the N-terminus, C-terminus and the M3/M4 loop. The alignment was used in conjunction with the GluClα crystal structure to guide mutagenesis. The alignment was created in CLC Main Workbench 7. Incorporation of an Asc1 restriction site (GRA) was used to insert fluorescent proteins into the M3/M4 loop of either the α1 or β2 subunit. Truncation of the M3/M4 loop of GABAA subunits were designed to mimic the shortened loop of GluClα (AGT) in the crystal structure. The location of M3 and M4 (red) were derived from alignments with GluClα combined with secondary structure prediction. (TIF) [file pone.0201210.s001.tif]

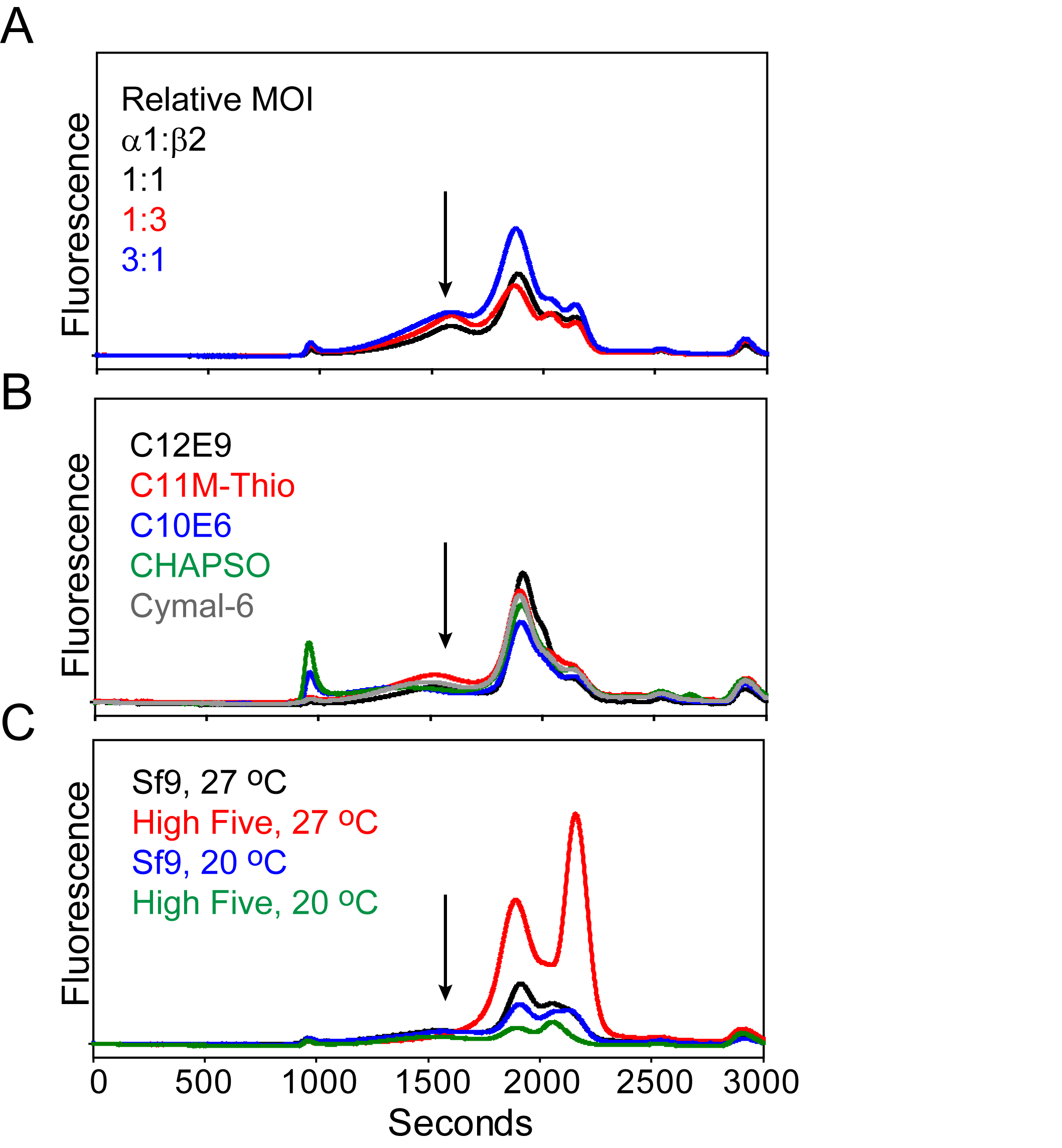

Supplement: S2 Fig — (A) Infection of Sf9 cells with two baculoviruses harboring the α1-EGFP and β2 subunits at different subunit ratios (relative MOI) does not improve expression as observed 72 hours post infection. (B) Screening a panel of detergents for whole-cell solubilization. (C) Infection of High Five cells or shifting cultures to lower temperatures does not improve receptor recovery. The FSEC traces show absolute fluorescence intensities and are plotted on the same scale as Fig 7C. The arrows point to the expected elution position of the receptor. (TIF) [file pone.0201210.s002.tif]

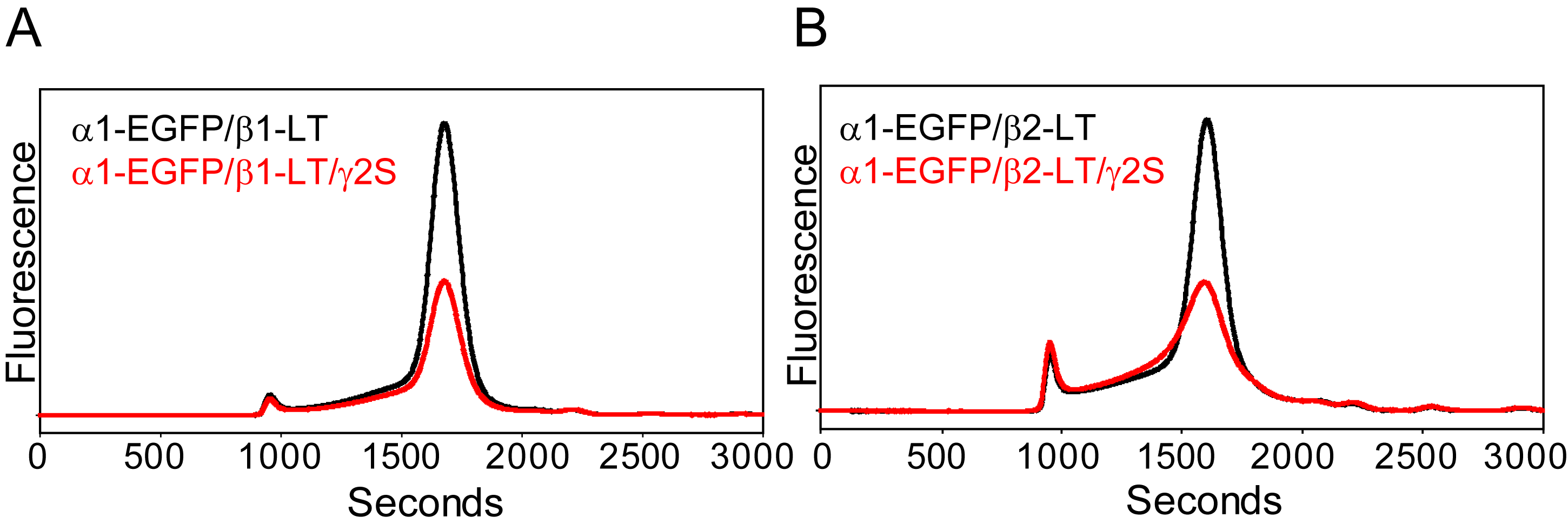

Supplement: S3 Fig — Incorporation of the γ2S subunit appears to reduce receptor expression levels relative to α1-EGFP/β1-LT (A) or α1-EGFP/β2-LT (B) at similar time points. (TIF) [file pone.0201210.s003.tif]

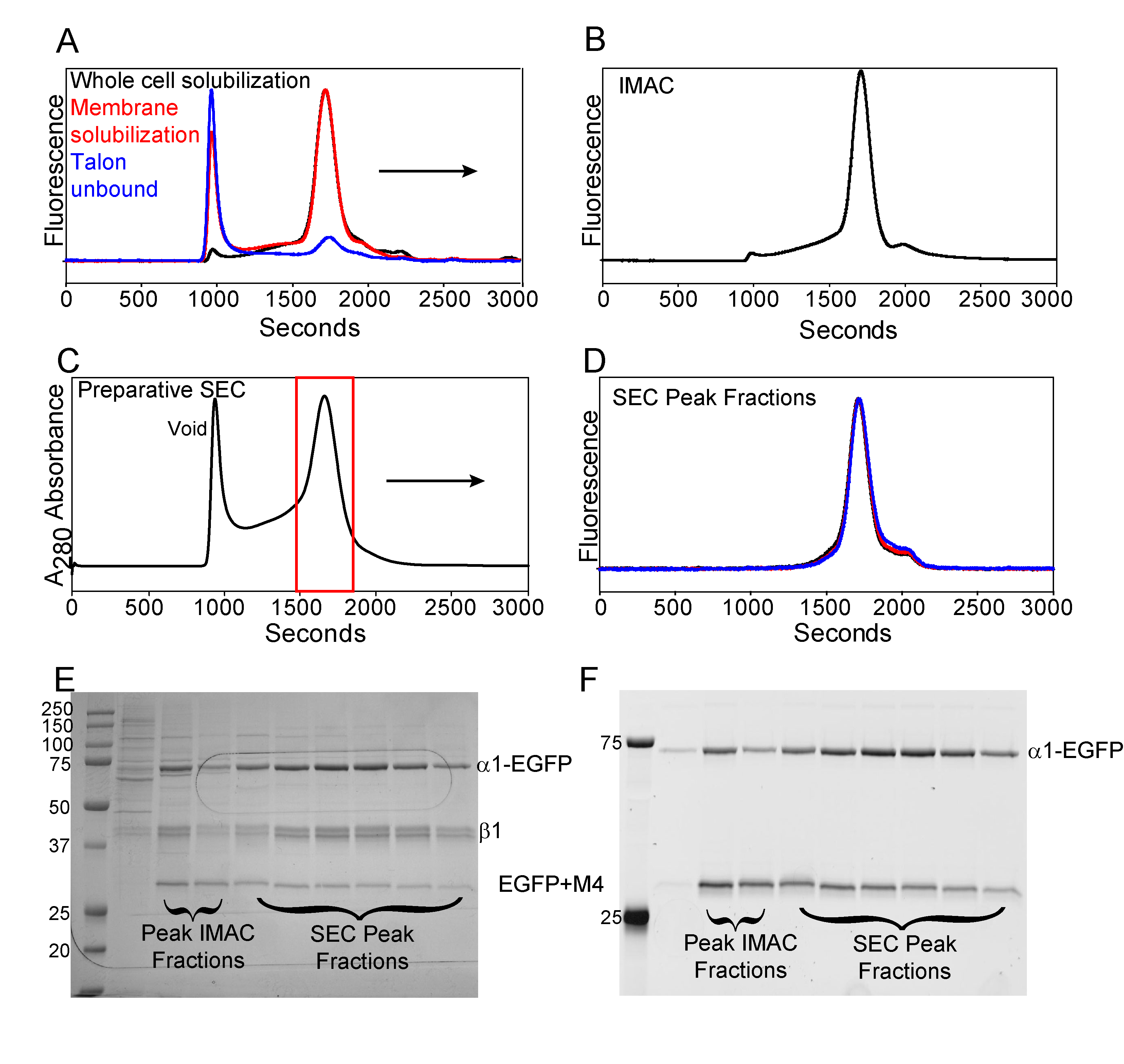

Supplement: S4 Fig — (A) The receptor demonstrates a similar FSEC profile whether solubilized from whole cells (black trace) or from the membrane fraction (red trace). The traces are normalized to emphasize similarities. Based on the FSEC analysis, approximately 50–55% (~3.3 mg) of the expressed receptor (6.4 L culture) was extracted from the membrane fraction. Of this material, 90% of the receptor was bound to Talon resin after three hours batch binding as reported by a depletion of receptor from the solution (blue trace). Approximately 3mg of receptor (0.5 mg/L or ~2 nmol/L) eluted from the resin (B), and was concentrated for preparative SEC (C). Peak fractions as identified by the red box in (C) are monodisperse by FSEC analysis (D). (E) SDS-PAGE analysis demonstrates the purity of the receptor as a function of purification steps. The first lane shows contaminants eliminated by a wash with 30 mM imidazole during IMAC purification, followed by two lanes showing elution fractions obtained from 250mM imidazole. The following six lanes show purity in fractions obtained across the SEC elution peak, which correspond to the FSEC analysis shown in (D). Notably, the presence of EGFP in the α1 M3/M4 loop enhanced proteolytic cleavage relative to non-fusion constructs, giving rise to a ~30 kDa band in (E). Confirmation that this band contained EGFP was made by in-gel fluorescence prior to fixing and staining (F). Although we did not explicitly determine the identity of this band, the migration position is consistent with EGFP plus the M4 α-helix. However, cleavage of the loop likely does not disrupt native associations since the purified receptor demonstrated monodispersity by FSEC analysis as shown in panel D. (TIF) [file pone.0201210.s004.tif]

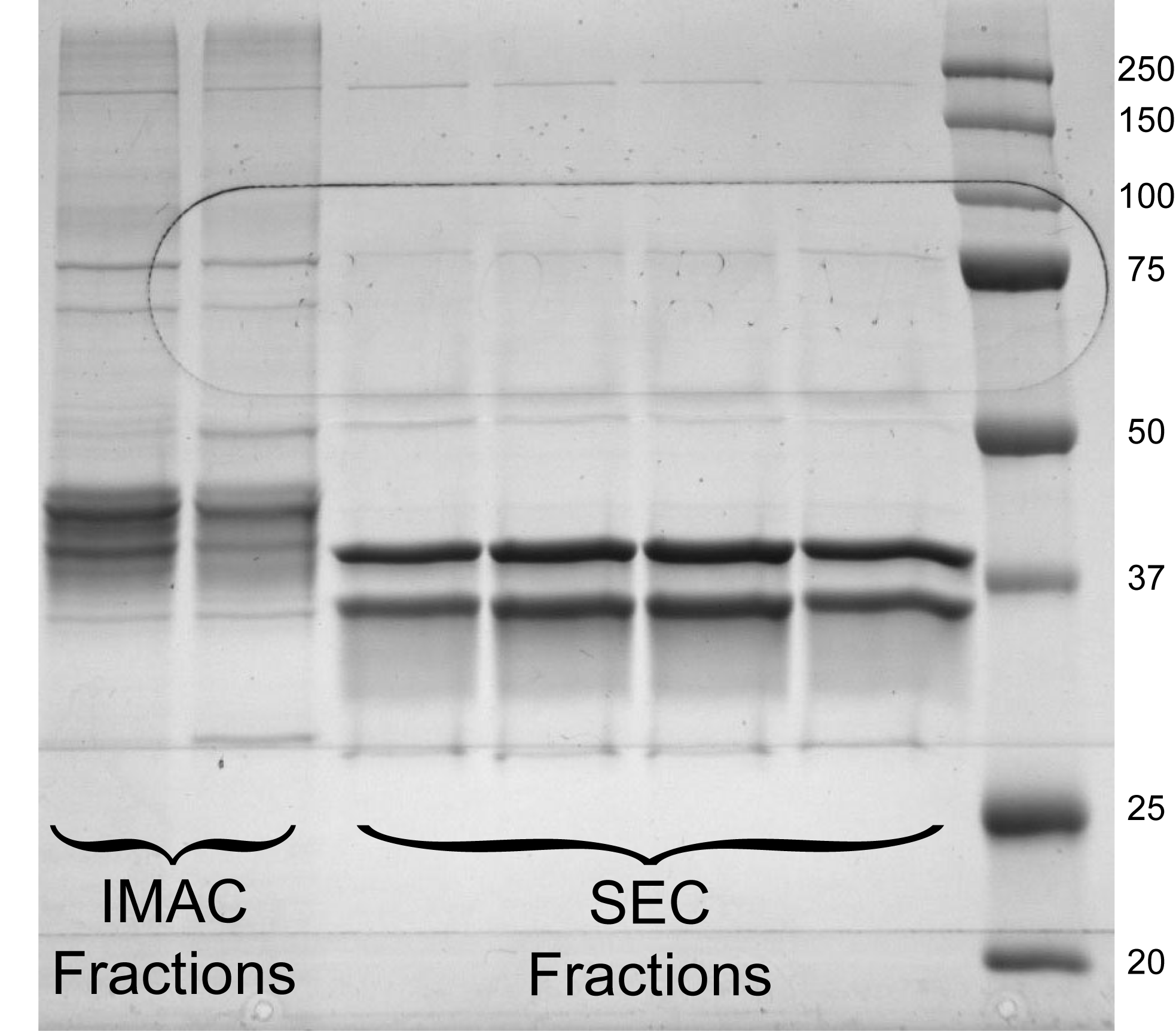

Supplement: S5 Fig — Following IMAC purification, the receptor was treated with EndoH (1:1, w/w) and EndoF3 (1:50, w/w) for two hours at room temperature at pH 6.5. After purification by SEC, the receptor was analyzed by SDS-PAGE and showed that treatment caused the diffuse bands seen in the IMAC fractions to collapse into a single prominent band for both subunits. (TIF) [file pone.0201210.s005.tif]
